# Inhibitory Effect and Mechanism of Chitosan–Ag Complex Hydrogel on Fungal Disease in Grape

**DOI:** 10.3390/molecules27051688

**Published:** 2022-03-04

**Authors:** Weizhong He, Yajuan Zhu, Yan Chen, Qi Shen, Zhenyu Hua, Xian Wang, Peng Xue

**Affiliations:** 1 Institute of Quality Standards & Testing Technology for Agro-Products, Xinjiang Academy of Agricultural Sciences, Urumqi 830091, China; hewei198112@126.com (W.H.); shenqi068@163.com (Q.S.); huakobe@163.com (Z.H.); wangxian_707@163.com (X.W.); 2 Key Laboratory of Agro-Product Quality and Safety of Xinjiang, Laboratory of Quality and Safety Risk Assessment for Agro-Products, Ministry of Agriculture and Rural Affairs, Urumqi 830091, China; 3 The Center for Disease Control and Prevention of Xinjiang Production and Construction Crops, Urumqi 830002, China; 4 Wenzhou Institute, University of Chinese Academy of Sciences, Wenzhou 325001, China; chenyan20@ucas.ac.cn

**Keywords:** silver, chitosan, hydrogel, antibacterial, grape

## Abstract

Hydrogel antibacterial agent is an ideal antibacterial material because of its ability to diffuse antibacterial molecules into the decayed area by providing a suitable microenvironment and acting as a protective barrier on the decay interface. The biocompatibility and biodegradation make the removal process easy and it is already widely used in medical fields. However, there have been few reports on its application for controlling postharvest diseases in fruit. In this study, the Chitosan–silver (CS–Ag) complex hydrogels were prepared using the physical crosslinking method, which is used for controlling postharvest diseases in grape. The prepared hydrogels were stable for a long period at room temperature. The structure and surface morphology of CS–Ag composite hydrogels were characterized by UV-Vis, FTIR, SEM, and XRD. The inhibitory effects of CS–Ag hydrogel on disease in grape caused by *P. expansum, A. niger,* and *B. cinerea* were investigated both *in vivo* and *in vitro*. The remarkable antibacterial activity of CS–Ag hydrogels was mainly due to the combined antibacterial and antioxidant effects of CS and Ag. Preservation tests showed that the CS–Ag hydrogel had positive fresh-keeping effect. This revealed that CS–Ag hydrogels can play a critical role in controlling fungal disease in grapes.

## 1. Introduction

The decay process of fruit is caused by infection and reproduction of microorganisms [1]. *Aspergillus* and *Penicillium* produce toxic secondary metabolites during postharvest of grape, which pose a high risk of disease for consumers; as such, a demand for natural, green, and safe antibacterial agents for the preservation of grapes is growing [2,3]. Hydrogel antibacterial agent is a synthetic or natural biological material, which is an ideal antibacterial material [4,5]. It diffuses antibacterial molecules into the decayed area by providing a suitable microenvironment and acts as a protective barrier at the decay interface [6]. The biocompatibility, biodegradation and self-healing properties make the removal process easy, and it is widely used in medical and agriculture [7,8,9].

Hydrogels based on chitosan (CS) have attracted considerable interest and have been widely used as antibacterial agents because of their non-toxic, biocompatible, renewable, and biodegradable features [10,11,12]. The broad-spectrum antibacterial activity of chitosan is related to its physical and chemical properties and is restricted by the types of microorganisms [13]. The antibacterial properties can be significantly increased when CS is blended with a silver ion (Ag^+^) or silver nanoparticles (AgNPs) [14]. However, the AgNPs are easy to agglomerate and oxidize when exposed to air, which affects antibacterial performance. In addition, direct application of AgNPs may also cause damage to the human liver [15]. One particular solution is to prevent the self-aggregation of Ag particles by embedding Ag composites into hydrogel frameworks [16,17]. Although Chitosan–silver (CS–Ag) conjugate materials as bacteriostatic agents have been successfully commercialized in the medical and pharmaceutical fields, the application in agriculture is not mature [18]. CS–AgNPs are used to make membranes, and the system is more inclined to a sol state than a gel, while the CS–Ag complex has the properties of rapid gelation and a stable gel structure at room temperature [7,8,9,10,11]. These unique properties make it easier to keep fruit fresh and for commercial applications. Furthermore, there are few reports on the direct application of CS–Ag complex hydrogels for controlling postharvest diseases in fruit.

In this communication, the CS–Ag hydrogels were prepared using the physical crosslinking method and characterized by UV-Vis, FTIR, SEM, and XRD. The inhibitory effect of CS–Ag gel against the fungal species during grape storage were investigated both *in vitro* and *in vivo*. Preservation tests showed that the CS–Ag hydrogel had a positive fresh-keeping effect. Furthermore, the inhibitory effect of the CS–Ag complex gel on fungi and the underlying mechanism of the CS–Ag gel against the fungi were explored.

## 2. Results and Discussion

### 2.1. Fungal Identification

Three fungal species were identified as *Penicillium expansum* (*P. expansum*), *Aspergillus niger* (*A. niger*) and *Botrytis cinerea* (*B. cinerea*) by Morphological and molecular identification. (Supplementary Materials, Figures S1 and S2).

### 2.2. CS–Ag Complex Hydrogel Characterization

**Figure 1:**
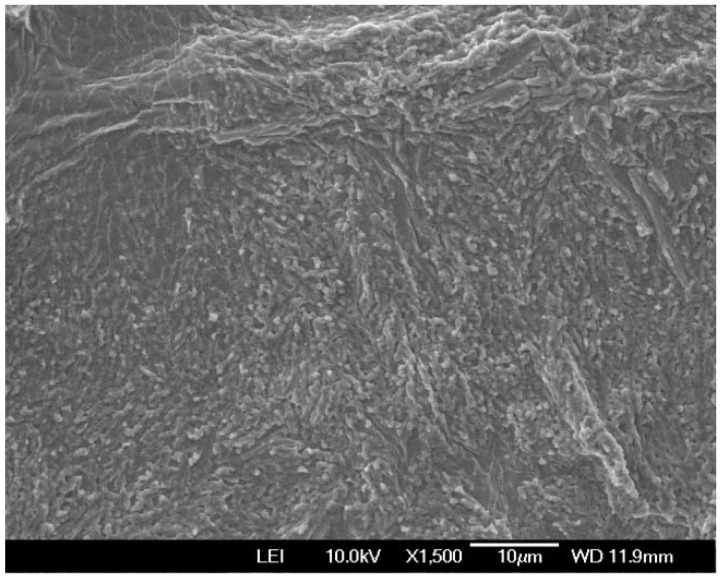
SEM image of the CS–Ag complex hydrogel.

The CS–Ag hydrogel had excellent gelation properties. The scanning electron microscopy (SEM) of the morphology of the CS–Ag hydrogel (Figure 1) revealed that the CS–Ag sample was composed of interconnected nanostructures, whereas the CS alone was much smoother (Supplementary Materials, Figure S3), indicating that the interwoven networks were cross-linked by Ag^+^ ions [19].

**Figure 2:**
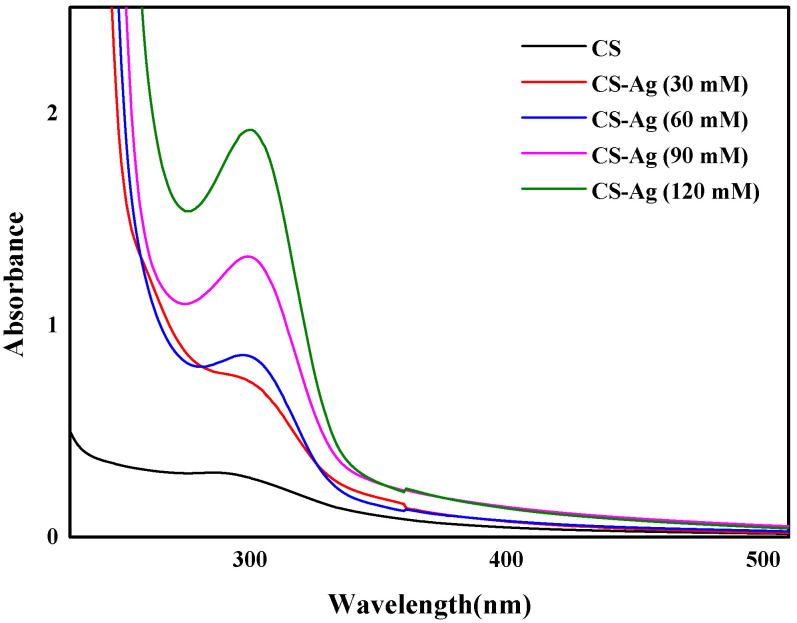
The concentration-dependent UV-Vis absorption spectra of CS–Ag hydrogel.

**Figure 3:**
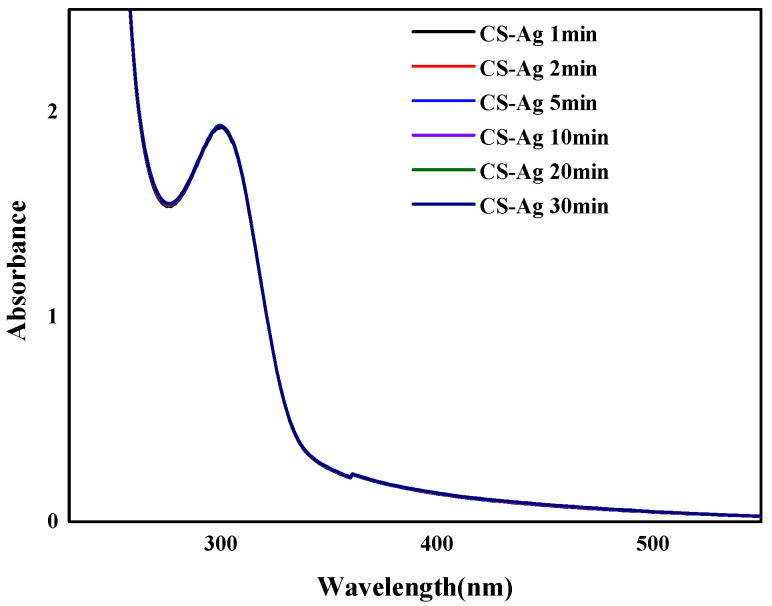
The time-dependent UV-Vis absorption spectra of CS–Ag (120 mM).

The Ag^+^ ions in the complexation with CS were proved by concentration-dependent UV-Vis absorption spectra (Figure 2). The CS solution transformed into a hydrogel when the Ag^+^ solution was gradually introduced. The intensity of the absorbance increased significantly with increasing Ag^+^ ion concentration, with the maximum absorption band undergoing a red shift from *λ* = 287 nm to 298 nm. This result was attributed to the coordination of the Ag^+^ ions (the metal, M) to the CS chains (the ligand, L), which have plenty of –O^−^ and –NH_2_ with abundant lone-pair electrons. The formation of the complex was described according to Lewis acid–base theory, where the acid is the acceptor for a pair of electrons donated by the base [20]. The time-dependent UV-Vis absorption spectrum (Figure 3) indicated that the process is not time dependent, which can be ascribed to the extremely fast complexation between the Ag^+^ ions and the CS polymer chains.

**Figure 4:**
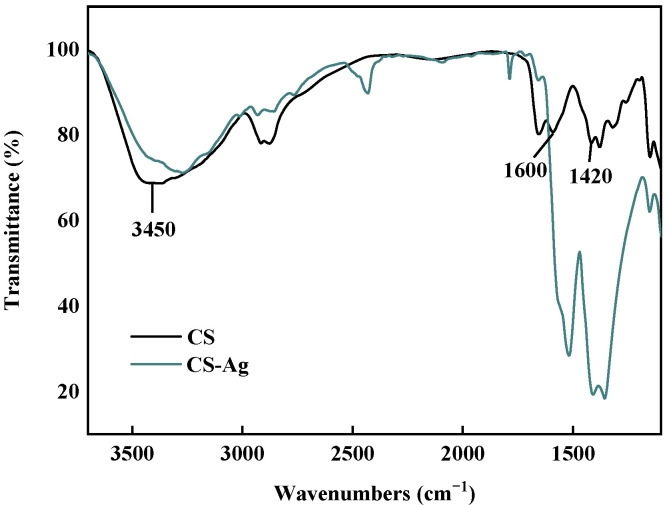
FTIR spectra of CS powders and CS–Ag xerogels.

The FTIR spectra of the CS powders and the CS–Ag xerogels were confirmed the complexation of CS and Ag^+^. The major evidence for the interaction of CS and Ag^+^ ions is the shift of the band at 3450 cm^−1^ (attributable to CS *v*OH and *v*NH absorptions) to 3420 cm^−1^ (Figure 4) [20]. Furthermore, from CS to CS–Ag xerogel the amide II band at 1600 cm^−1^ underwent a blue shift, and the C-N band at 1420 cm^−1^ a red shift. These shifts revealed that both -OH and -NH_2_ groups in the CS chains took part in the coordination complexation which promoted the rapid gel-network formation [21]. 

**Figure 5:**
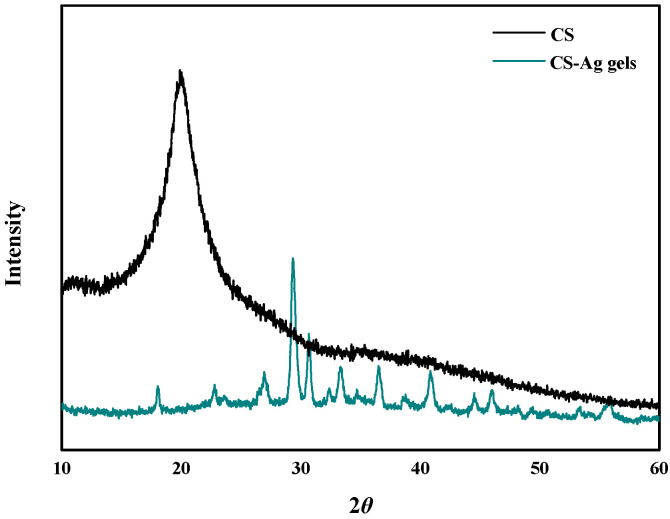
XRD pattern of the CS powders and CS–Ag xerogels.

The XRD patterns of the CS–Ag xerogel (Figure 5), the intensity of the peak at 2*θ* = 20° for the crystal form of the pure CS powders decreased significantly and only a much broader, less intense peak at 2*θ* = 30° was detected for the CS–Ag xerogels. This change in the XRD spectrum is as a result of the complexation between the Ag^+^ ions and the CS chains through -OH and -NH_2_ binding sites, efficiently reducing the hydrogen bonding and destroying the crystal structure of the CS chains [21]. These results were in agreement with those of UV-Vis and FTIR.

### 2.3. Inhibition of CS–Ag Complex Hydrogel on Fungal Disease In Vitro

**Figure 6:**
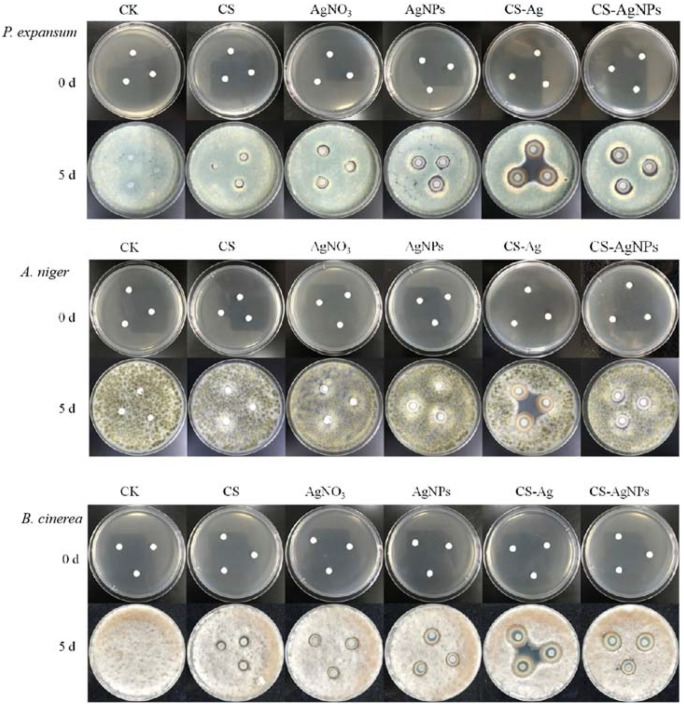
The antibacterial effect of different concentrations of CS–Ag xerogels bacteriostatic agent on colony growth of *P. expansum, A. niger* and *B. cinerea.* (0.62 wt% CS–Ag hydrogel, CS 1.7 mg·mL^−1^, Ag^+^ 4.5 mg·mL^−1^; 0.62 wt% CS–AgNPs sol, CS 1.7 mg·mL^−1^, AgNPs 4.5 mg·mL^−1^; CS 1.7 mg·mL^−1^; AgNO_3_ 4.5 mg·mL^−1^; AgNPs 4.5 mg·mL^−1^).

The antibacterial results of CS–Ag gel and CS–AgNPs hybrid sol on *P. expansum, A. niger* and *B. cinerea* were shown in Figure 6. The CS–Ag gel revealed a remarkable inhibitory effect on all tested fungi. In the absence of CS–Ag gel, colonies expanded over time. Compared with the control (CK) groups, *P. expansum, A. niger* and *B. cinerea* could not grow and reproduced on CS. The edge of the antibacterial area on CS was not obvious. Ag^+^ had a certain inhibitory effect on three fungi, which was slightly inferior than AgNPs. The antibacterial effect of CS–AgNPs sol on *P. expansum, A. niger* and *B. cinerea* were similar to that of AgNPs. CS–Ag hydrogel showed obvious antibacterial area to all tested fungi.

### 2.4. Effect of CS–Ag Complex Hydrogel on Grape Disease In Vivo

**Figure 7:**
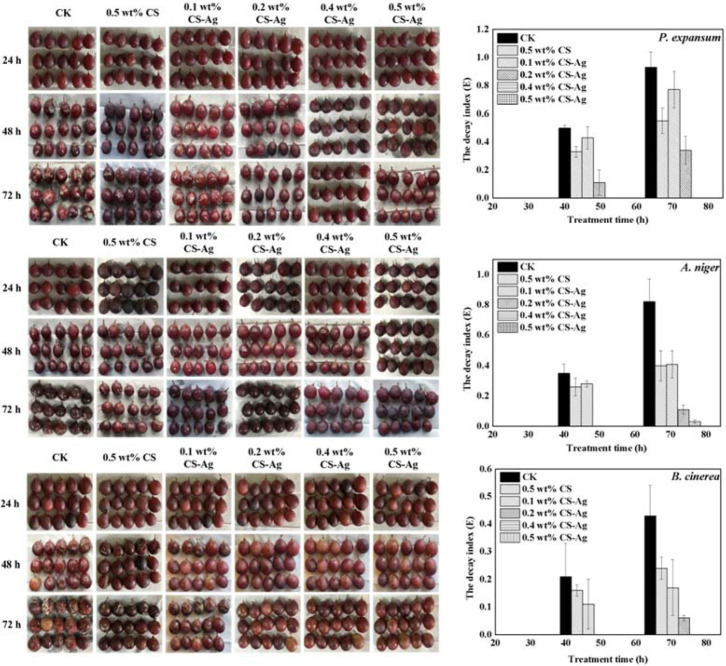
Antibacterial effect of CS–Ag gel bacteriostatic agents on fungal disease caused by *P. expansum, A. niger,* and *B. cinerea* in grape. Vertical bars represented standard deviations of the means.

The different concentrations of CS–Ag gel antibacterial agents (0.1, 0.2, 0.4, 0.5 wt%) were selected to treat grapes that had been inoculated with fungi (Figure 7). The results show that inverse correlation was found between fungal growth and CS–Ag hydrogel concentration. CS–Ag gel can significantly inhibit the fungal diseases caused by *P. expansum*, *A. niger* and *B. cinerea*. CS–Ag gel could delay the onset in grapes, reduce the severity of grape diseases and the grape decay index. The CK samples which were sprayed with a suspension of *P. expansum*, *A. niger,* and *B. cinerea* begun to decay at 48 h. The effect of the 0.1 wt% CS–Ag gel treatment group was similar to that of the 0.5 wt% CS group. Although decay had occurred, the decay index was significantly lower than that of the CK group The experimental data expressed that 0.4 wt% CS–Ag gel agent has obvious inhibitory effect on *P. expansum* and *B. cinerea*., and 0.2 wt% CS–Ag gel could inhibit *A. niger*. The occurrence of fungal diseases in grape were completely controlled within 72 h, and the disease index was 0. Compared with the CK group, the decay index of *P. expansum*, *A. niger,* and *B. cinerea* disease decreased by 100%, 96.34%, and 100%, respectively.

In order to investigate the effects of CS on grape preservation, the grapes were treated with different concentrations of CS–Ag gel and stored at room temperature for 40 days (d). The decay rate and weight loss rate of grape greatly affect its commercial value and edible taste. An inverse correlation was found between decay rate and CS–Ag gel concentration (Supplementary Materials, Figure S6). As the increase of time, the weight loss rate of the CK group was significantly higher than the other groups and the 0.5 wt% CS–Ag gel agent and the 0.5 wt% CS solution were lower than others (Supplementary Materials, Figure S7). This may indicate that CS treatment is easy to form a film on the fruit surface, which increases the peel thickness and block part of the peel pores. To some extent, it can reduce the transpiration of water in grapes and retain the water [9,14]. The experiment of total soluble solid (TSS), titratable acid, and vitamin C content indicated that 0.2 wt% CS–Ag gel treatment could delay the decrease of nutrient loss in fruit and maintain the content of the grape’s physiological quality at a certain level. (Supplementary Materials, Figures S8–S10).

In conclusion, CS–Ag gel agent can not only control grape fungal diseases well, but also preserve grape storage quality.

### 2.5. Effect of CS–Ag Complex Hydrogel on Fungal Morphology

**Figure 8:**
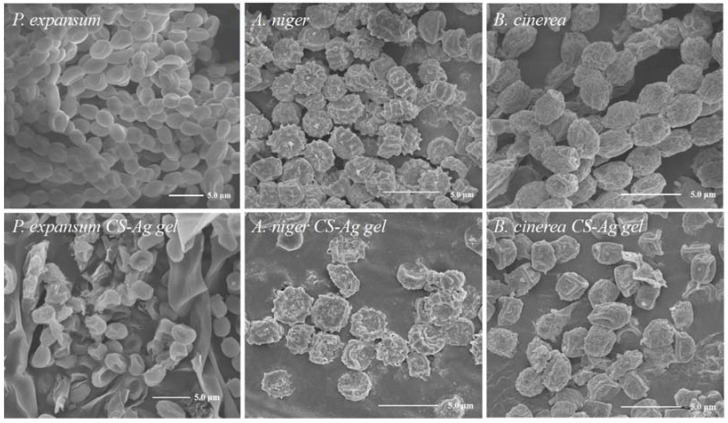
Effect of CS–Ag on surface microstructure of *P. expansum, A. niger* and *B. cinerea*. (0.62 wt% CS–Ag hydrogel, CS 1.7 mg·mL^−1^, Ag^+^ 4.5 mg·mL^−1^).

We used scanning electron microscopy (SEM) to obtain a direct view of the morphology of the fungi. As shown in Figure 8, the SEM image revealed that treatment with the CS–Ag gel antibacterial agent had a great influence on the morphology of the cell wall of conidia such as *P. expansum*, *A. niger* and *B. cinerea*, and the structure of the cell wall was damaged by folds or even broken, resulting in the leakage of cell protoplasm. Integrated with the literature reports, the destruction of the structure of the fungus by the CS–Ag gel antibacterial agent may be caused by the additive effect of CS and Ag^+^ [13]. Both chitosan and Ag^+^ have a positive charge, which can interact with pathogen’s negative charge on the surface of the cell membrane and change the permeability of cell membrane which could cause cell material leakage and reduce the cell activity which leads to cell membrane and fungal DNA damage [22,23].

The CS–Ag hydrogel changes the morphology of mycelia and destroys the integrity of the cell membrane, which may affect the permeability of plasma membrane leading to imbalance of intracellular osmotic pressure and leakage of cytoplasmic contents [24,25]. Moreover, the hydroxyl groups in antibacterial compounds can form hydrogen bonds with active enzymes, resulting in deactivation and blocked enzymatic reactions; these may lead to cell necrosis and inhibit the growth and reproduction of fungi to control grape mycosis. These additive effect of CS and Ag^+^ can probably lead to cell necrosis easily [26,27,28,29].

### 2.6. Residue of Silver in Grape

**Figure 9:**
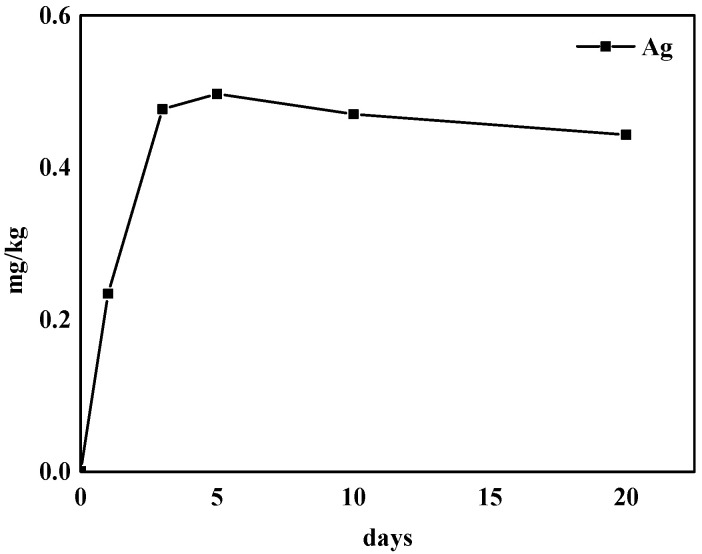
The change of Ag residues in grape after CS–Ag complex hydrogel preservation.

Since chitosan is derived from natural substances and has the characteristics of being non-toxic and having good water-solubility, the residues of CS–Ag after washing mainly refer to Ag^+^. The Ag^+^ concentration of the grapes after preservation increases with each day, and the saturation value reached after 5 days is about 0.49 mg/kg (Figure 9). After that, the Ag^+^ concentration tends to remain unchanged with the increase of storage time.

However, there are no adequate data with which to derive a health-based guideline value for silver in drinking-water and food [30]. The reference value is underpinned by the prior assessment that 10 g of ingested silver can be considered a human NOAEL (No Observed Adverse Effect Level) [31]. Therefore, assuming grape intake of 1 kg/day, 0.49 mg/kg is a concentration in grape that would give a total dose over 28 years of half this NOAEL. These could be tolerated in such cases without risk to health.

## 3. Materials and Methods

### 3.1. Fruit Material and Chemical Material

Grape (Red Globe) were purchased from Tulufan, Xinjiang, China. All fruits were free from mechanical damage or infection. The surface of the selected fruits was disinfected with 75% ethanol for 5 min, washed twice with sterile water, and air-dried.

Low molecular weight chitosan (85% deacetylated) and agar powder (bacteriological grade) were procured from Sigma-Aldrich (St. Louis, MO, USA). Silver nitrate (AgNO_3_, 99.8%; Energy Chemical, Shanghai, China), acetic acid (glacial, 99 100%; Sinopharm, Beijing, China), sodium citrate (95%; Sinopharm), and sodium hydroxide (NaOH, Sinopharm) 4-nitrophenol (95%; Merck, Darmstadt, Germany) were used as received without further purification. All other chemicals used in this study were of analytical grade.

### 3.2. Fungal Isolation, Identification, Culture, and Spore Suspension

This procedure references previous literature [32]. The decayed grape was washed with sterile water, the filtrate was collected and diluted, and then 100 μL diluted solution was uniformly spread on a petri dish containing potato dextrose agar (PDA) medium and cultured at 28 °C for 7 d. The fungal isolates were purified three times on PDA. 

The major isolates were obtained and characterized using macroscopic and microscopic observation methods. Le Lay’s method was used for genotyping (2016), and the obtained sequences were subjected to a comparison with the GenBank database using the Basic Local Alignment Search Tool (BLAST; http://www.ncbi.nlm.nih.gov/BLAST, accessed on 21 June 2021). 

The isolated fungi were separately cultured on PDA at 28 °C for 7 d. Spore suspensions were obtained by rinsing the cultures with sterile water containing 0.05% (*v/v*) Tween-80 and then adjusted to 1 × 10^9^ CFU/L. 

### 3.3. Characterization

The UV-Vis spectrum was recorded on Shimadzu UV-2550 spectrometer (Kyoto, Japan) (Details in Section 3.4). Fourier transform infrared spectroscopy (FTIR) was carried out on a Bruker EQUINOX-55 (Karlsruhe, Germany). X-ray diffraction (XRD) was carried out using Bruker D8 Advance (Billerica, MA, USA). Xerogel was freeze-dried for 24 h before performing the FTIR and XRD measurements to decrease the influence of moisture content. Scanning electron microscopy (SEM) images were obtained from the field emission SEM (SU8010, Hitachi, Tokyo, Japan) (Details in Section 3.8). Transmission electron microscopy (TEM) was taken using a Hitachi H-600 (Tokyo, Japan) operated at 80 kV. The size distribution measurement was recorded on a laser particle size analyzer (LDPSA) Malvern Zetasizer Nano S90 (Malvern Panalytical Ltd., Malvern, UK) Inductively coupled plasma-mass spectrometry (ICP-MS) was recorded on ThermoFisher iCAP Q (Waltham, MA, USA) (Details in Section 3.9).

### 3.4. Preparation of CS–Ag Complex Hydrogel and CS–AgNPs Sol

Chitosan–Ag Complex Hydrogel: The 1 mL CS–Ag hydrogel was prepared by adding 100 μL 0.2 M NaOH to 800 μL 0.5 wt% CS solution (in 1% acetic acid), followed by adding 100 μL freshly prepared 0.3 M AgNO_3_ solution with vigorous shaking for 2 s. The Critical Gelation Concentration (CGC) of the hydrogel was visually recognized by the reversed vial test method (Supplementary Materials, Figure S4). Confirmation that in this case the CS–Ag complex systems have been obtained will be described below based on SEM, UV-Vis, FTIR, and XRD measurements.

Chitosan–AgNPs Sol: The 0.3 M AgNPs sol was prepared by adding 10 mL 3.0 M AgNO_3_ to 80 mL water, followed by adding 10 mL 3.0 M sodium citrate standard solution with vigorous shaking, and heated in the microwave oven for 3 min. The AgNPs diluted solution was dropped onto the copper mesh, and the morphology was observed by TEM (Supplementary Materials, Figure S5). TEM results revealed that the formation of AgNPs with sizes ranging from 80 to 110 nm. The result of size distribution of AgNPs is close to TEM measurements (Supplementary Materials, Figure S6).

The 1 mL CS–AgNPs Sol was prepared by adding 100 μL 0.2 M NaOH to 800 μL 0.5 wt% CS solution (in 1% acetic acid) followed by adding 100 μL 0.3 M AgNPs sol with vigorous shaking.

### 3.5. Zone of Inhibition Test

All experimental operations are carried out on a clean workbench.

#### 3.5.1. Preparation of Fungal Suspension

Pick up 3 needles of pathogenic fungi strains into the test tube containing 10 mL sterile water, and then shake the test tube well to make 10^−1^ fungi suspension. 

#### 3.5.2. Antibacterial Experimental Method

Preparation of bacteriostatic tablets: Prepare the qualitative filter paper into a circular paper with a diameter of 6 mm with a punch, then put these filter papers into an autoclave at 121 °C for 20 min, then dry for later use. Immerse the sterilized and dried 6 mm filter paper disc into different bacteriostatic solutions.

Fungal inoculation: inoculate 500 µL of the fungal suspension on the culture medium, mix evenly, then cover the petri dish and dry it at room temperature for 5 min.

Put the filter paper that has been treated with antibacterial agent gently on the medium coated with bacteria by using sterilized tweezers, and put 3 pieces on each medium. The distance between the centers of each filter paper is more than 30 mm, and the distance from the edge of the petri dish is more than 20 mm. The filter paper was tightly attached to the medium, covered the petri dish, and placed at 28 °C for 5 d observation.

After pasting the filter paper, gently press the filter paper with tweezers to make it tightly adhere to the culture medium and cover the culture dish, place it in a 28 °C incubator and culture for 5 d. Measure the diameter of the bacteriostatic circle with a vernier caliper and record the results.

### 3.6. Control of CS–Ag Hydrogel on Fungal Disease In Vivo

In order to ensure pathogenicity, the grapes were uniformly sprayed with suspensions of the tested fungal spores and dried. The grapes were soaked in CS–Ag hydrogel at different concentrations, and then placed in a sealing bag. All samples were cultured at 20 ± 2 °C and 70 ± 10% relative humidity in the incubator. Surface decay on the grapes was recorded on a regular basis. All treatments were performed in triplicate and the entire experiment was repeated two times.

The decay index (E) was calculated by scoring the total decayed area on each fruit surface: class 0, no visible decay; class 1, decay accounts for less than 25% of the surface area; class 2, decay accounts for 25–50% of the surface area; class 3, decay accounts for 50–75% of the surface area; and class 4, decay accounts for more than 75% of the surface area. The decay index (E) was calculated using the following formula:

(1) $$\mathrm{The}\;\mathrm{decay}\;\mathrm{index}\;\left(E\right)=\frac{\sum \left(\mathrm{the}\;\mathrm{number}\;\mathrm{of}\;\mathrm{decayed}\;\mathrm{fruits}\;\mathrm{in}\;\mathrm{each}\;\mathrm{class}\;\times \mathrm{decay}\;\mathrm{scale}\right)}{\mathrm{the}\;\mathrm{total}\;\mathrm{number}\;\mathrm{of}\;\mathrm{treated}\;\mathrm{fruits}\times \;\mathrm{the}\;\mathrm{decay}\;\mathrm{scale}}\;\;\;\;$$

### 3.7. Preservation Test of CS–Ag Hydrogel

The fresh grapes were divided into seven parts and separately soaked in 0.5% Chitosan, 0.1% CS–Ag Hydrogel, 0.2% CS–Ag Hydrogel, 0.4% CS–Ag Hydrogel, 0.5% CS–Ag Hydrogel, CK group with no processing. The soaked grapes were sampled regularly at room temperature (temperature 20 ± 2.0 °C, relative humidity 20 ± 5%), the storage quality was evaluated by determining the weight loss rate, decay rate, soluble solids, titratable acid, and vitamin C content. All indicators were determined, and each treatment was repeated three times. The vertical bar represents the standard deviation of the mean.

The decay rate:

(2) $$\mathrm{The}\;\mathrm{decay}\;\mathrm{rate}\;=\frac{\mathrm{the}\;\mathrm{number}\;\mathrm{of}\;\mathrm{decay}\;\mathrm{grape}}{\mathrm{the}\;\mathrm{number}\;\mathrm{of}\;\mathrm{grape}}\times 100\%\;\;\;$$

Weight loss rate: The weight of grapes was weighed by weighing method before and after storage, and the weight loss rate was calculated.

(3) $$\mathrm{Weight}\;\mathrm{loss}\;\mathrm{rate}\;=\frac{\mathrm{the}\;\mathrm{weight}\;\mathrm{difference}\;\mathrm{of}\;\mathrm{grape}\;\mathrm{before}\;\mathrm{and}\;\mathrm{after}\;\mathrm{storage}}{\mathrm{the}\;\mathrm{weight}\;\mathrm{of}\;\mathrm{grape}\;\mathrm{before}\;\mathrm{storage}}\times 100\%$$

Total soluble solid (TSS): The method of refractometer in NY/T 2637-2014 was used to determine the content of soluble solids in grape by using a hand-held digital refractometer.

Titratable acid content: The acid-base titration method was used. Take appropriate numbers of grapes, homogenate, and add 30 mL distilled water into triangle bottle. Place in a water bath for 30 min, cool, and filter. A total of 10 mL filtrate was added with phenolphthalein indicator and titrated to micro red with sodium hydroxide solution.

Vitamin C content: The method of 2,6-dichloroindophenol titration in GB5009.86-2016 was used to determine vitamin C content.

### 3.8. Morphology of Fungal

The analysis of differences in the micro-morphological structures between the CS–Ag and CK groups for the tested fungi were examined using a scanning electron microscope (SEM). For that a 10 μL drop of each sample was deposited on a silicon slide, dried, and sputter-coated with gold film in a sputter coater.

### 3.9. Residue of Silver in Grape

Remove impurities from fresh grapes, take the edible part, wash and dry, and make into a homogenate. Weigh 3.0 g of the homogenized sample into a polytetrafluoroethylene digestion tank, then add 6 mL nitric acid and 1 mL hydrogen peroxide. After digestion is complete, dilute the digestion solution to 50 mL with ultrapure water before testing. The blank experiments are conducted simultaneously. All of the experiments were repeated three times. ICP-MS was used to detect Ag^+^ residue in grape after CS–Ag complex hydrogel preservation.

$$X=\frac{\left(c-c0\right)\times V\times f}{m\times 1000}$$

X—the silver content in sample (mg/kg);

c—determination value of silver in sample solution (ng/mL);

c0—determination value of silver in sample blank solution (ng/mL);

V—constant volume of sample (mL);

f—dilution ratio of sample;

m—the weight of sample (g).

## 4. Conclusions

In summary, the CS–Ag complex hydrogels were prepared rapidly using the physical crosslinking method and were stable for a long period at room temperature. Chitosan and silver coordination was confirmed by UV-Vis, FTIR, SEM, and XRD. CS–Ag hydrogel could effectively control disease in grapes and strongly inhibits the growth of *P. expansum*, *A. niger* and *B. cinerea* in both in vitro and in vivo studies. The antibacterial performance of CS–Ag complex can be explained as an additive effect of chitosan and silver. Chitosan could stabilize the Ag+ and prevent Ag+ oxidization and agglomeration. It can enhance binding to the negative charges present at the cell surface [27]. Although the exact mechanism in which CS–Ag operate to cause antimicrobial effect is not clearly known. One possible antibacterial mechanism involves CS–Ag being adsorbed on the surface of the cell membrane to form a layer of a polymer film which changes the selective permeability of the cell membrane and prevents the transport of nutrients into the cell [28]. Meanwhile, the interaction of CS–Ag with the bacterial cell wall and subsequent penetration causes structural changes in the cell membrane [29]. Furthermore, the preservation test showed that the CS–Ag hydrogel had positive fresh-keeping effect. The use of CS–Ag complex hydrogel can not only improve food safety by eliminating fungal spread but also leave no detectable residues after simple water washing. CS–Ag complex hydrogel improves grape safety and can be used as an alternative fungistatic agent for fruit to control fungal disease.

## Data Availability

The data presented in this study are available on request from the corresponding author.

## Supplementary Materials

The following are available online, Figure S1: The image of dominant pathogenic fungi isolated from grape. (a: *P. expansum*, b: *A. niger* and c: *B. cinerea*.), Figure S2: Identification of dominant pathogenic fungi isolated from grape (a: *P. expansum*, b: *A. niger* and c: *B. cinerea*.), Figure S3: SEM image of the CS gel, Figure S4: The CGC of CS–Ag complex hydrogel, Figure S5: TEM image of AgNPs, Figure S6: The size distribution of AgNPs; Figure S7: CS–Ag hydrogel effect on decay rate of grape during postharvest, Figure S8: CS–Ag hydrogel effect on weight loss rate of grape during postharvest, Figure S9: CS–Ag hydrogel effect on soluble solids of grape during postharvest, Figure S10: CS–Ag hydrogel effect on titratable acids of grape during postharvest, Figure S11: CS–Ag hydrogel effect on Vitamin C of grape during postharvest.

## Sample Availability

Samples of the compounds are available from the authors.
