# Supplementary material for: Inhibitory Effect and Mechanism of Chitosan–Ag Complex Hydrogel on Fungal Disease in Grape
Source: Molecules. 2022 Mar 4;27(5):1688. doi: 10.3390/molecules27051688 (PMC8911985; doi:10.3390/molecules27051688)
Supplement: Supplementary file 1 [file molecules-27-01688-s001.zip › molecules-1531094-supplementary.pdf]

Inhibitory Effect and Mechanism of Chitosan–Ag Complex Hydrogel on Fungal Disease in Grape

Weizhong He [1,2], Yajuan Zhu [3,*], Yan Chen [4], Qi Shen [1,2], Zhenyu Hua [1,2], Xian Wang [1,2], and Peng Xue [1,2,*]

[1] Institute of Quality Standards & Testing Technology for Agro-Products, Xinjiang Academy of Agricultural Sciences, Urumqi 830091, China; hewei198112@126.com (W.H.); shenqi068@163.com (Q.S.); huakobe@163.com (Z.H.); wangxian_707@163.com (X.W.)

[2] Key Laboratory of Agro-Product Quality and Safety of Xinjiang, Laboratory of Quality and Safety Risk Assessment for Agro-Products, Ministry of Agriculture and Rural Affairs, Urumqi 830091, China

[3] The Center for Disease Control and Prevention of Xinjiang Production and Construction Crops, Urumqi 830002, China

[4] Wenzhou Institute, University of Chinese Academy of Sciences, Wenzhou 325001, China; chenyan20@ucas.ac.cn.

* Correspondence: zhuyajuan1988@126.com (Y.Z.); xuepeng416@126.com (P.X.)

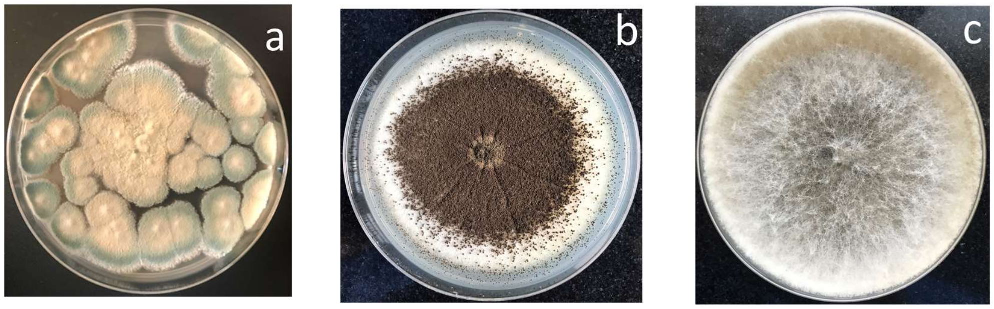

(**a**) (**b**) (**c**)

Figure S1. The image of dominant pathogenic fungi isolated from grape. ((**a**): *P. expansum*, (**b**): *A. niger* and (**c**): *B. cinerea*.).

a ITS1, Serial number:
TTGCGGTTACCCTACCTACTTCTGGTATCCCCCACTCCCATGGTGTGACGGGCGGTGTG-
TACAAGACCCGGGAACGTATTCACCGCAGTATGCTGACCTGCGATTACTAGCGAT-
TCCGACTTCATGCACTCGAGTTGCAGAGTGCAATCCGGACTACGATCGGTTTTGTGA-
GATTGGCTCCGCCTCGCGGCTTGGCTACCCTCTGTACCGACCATTGTATGACGTGTGAA-
GCCCTGGTCATAAGGGCCATGAGGACTTGACGTCATCCCCACCTTCCTCCGGCTTGTCACCGG-
CAGTCTCATTAGAGTGCCCAACCGAATGATGGCAACTAATGACAAGGGTTGCGCTCGTTGCGG-
GACTTAACCCAACATCTCACGACACGAGCTGACGACAGCCATGCAGCACCTGTGTTAC-
GGCTCCCGAAGGCACTCCTCCGTCTCCGGAGGATTCCGCACATGTCAAAAC-
CAGGTAAGGTTCTTCGCGTTGCATCGAATTAATCCACATCATCCACCGCTT-
GTGCGGGTCCCCGTCAATTCCTTTGAGTTTTAATCTTGCGACCG-
TACTCCCCAGGCGGTCAATTTCACGCGTTAGCTACGCTACCAAGCAATCAAGTT-
GCCCAACAGCTAATTGACATCGTTTAGGGCGTGGACTACCAGGGTATCTAATCCTGTTTGCTACCCACGCTT

a ITS4, Serial number:
TCGGACATGAACGTCAGTGTTATCCCAGGAGGCTGCCTTCGCCATCGG-
TATTCCTCCACATCTCTACGCATTTCACTGCTACACGTGGAATTCCAC-
CTCCCTCTGACACACTCGAGTCACCCAGTTCAGAACGCAGTTCCCGGGTTGAGCCCGGGGAT-
TTCACATCCTGCTTAAGTAACCGTCTGCGCCCGCTTTACGCCCAGTAATTCCGATTAAC-
GCTCGCACCCTACGTATTACCGCGGCTGCTGGCACGTAG-
TTAGCCGGTGCTTATTCTTCAGGTACCGTCATCGGCCGCCGATATTGG-
CAACAGCCTTTTCTTCCCTGACAAAAGTCCTTTACAACCCGAAGGCCTTCTTCAGACACGCGG-
CATGGCTGGATCAGGCTTGCGCCCATTGTCCAAAATTCCCCACTGCTGCCTCCCGTAGGAG-
TCTGGGCCGTGTCTCAGTCCCAGTGTGGCGGATCATCCTCTCAGACCCGC-
TACTGATCGTCGCCTTGGTGGGCCTTTACCCCGCCAACCAGCTAATCAGATATCGGCCGCTCG-
GATAGCGCAAGGCCCGAAGGTCCCCTGCTTTCCCTCTCAAGACGTATGCGG-
TATTAGCTGATCTTTCGATCAGTTATCCCCCGCTACCCGGTACGTTCCGA-
TATGTTACTCACCCGTTCGCCACTCGCCACCCGAGAAGCAAGCT

	Description	Scientific Name	Max Score	Total Score	Query Cover	E value	Per. Ident	Acc. Len	Accession
☑	Penicillium fimorum isolate 2011F12 small subunit ribosomal RNA gene, partial sequence; internal transcribed sp...	Penicillium fimorum	1026	1026	100%	0.0	100.00%	588	MT558942.1
☑	Penicillium robsamsonii isolate 2011F1 internal transcribed spacer 1, partial sequence; 5.8S ribosomal RNA gene...	Penicillium robsa...	1026	1026	100%	0.0	100.00%	574	MT558937.1
☑	Penicillium polonicum isolate 2010F27 internal transcribed spacer 1, partial sequence; 5.8S ribosomal RNA gene ...	Penicillium polon...	1026	1026	100%	0.0	100.00%	575	MT558934.1
☑	Penicillium italicum isolate 2010F18 internal transcribed spacer 1, partial sequence; 5.8S ribosomal RNA gene an...	Penicillium italicum	1026	1026	100%	0.0	100.00%	564	MT558932.1
☑	Penicillium expansum isolate 2010F13 internal transcribed spacer 1, partial sequence; 5.8S ribosomal RNA gene...	Penicillium expa...	1026	1026	100%	0.0	100.00%	562	MT558929.1

b ITS1, Serial number:
TGGSTTCGCTTCGTAGGTGACCTGCGGAGGATCATTACCGAGTGCGGGTCCTTT-
GGGCCCAACCTCCCATCCGTGTCTATTATACCCTGTTGCTTCGGCGGGCCCGCCGCTT-
GTCGGCCGCCGGGGGGGCGCCTTTGCCCCCCGGGCCCGTGCCCGCCGGAGACCCCAACAC-
GAACACTGTCTGAAAGCGTGCAGTCTGAGTTGATTGAATGCAATCAG-
TTAAAACTTTCAACAATGGATCTCTTGGTTCCGGCATCGATGAAGAACGCAGCGAAATGCGA-
TAACTAATGTGAATTGCAGAATTCAGTGAATCATCGAGTCTTTGAACGCACATT-
GCGCCCCCTGGTATTCCGGGGGGCATGCCTGTCCGAGCGTCATTGCTGCCCTCAA-

GCCCGGCTTGTGTGTTGGGTCGCCGTCCCCCTCTCCGGGGGGAC-

GGGCCCGAAAGGCAGCGGCGGCACCGCGTCCGATCCTCGAGCGTATGGGGCTTT-

GTCACATGCTCTGTAGGATTGGCCGGCGCCTGCCGACGTTTTCCAACCATTTTTTCCAGGTT-

GACCTCGGATCAGGTAGGGATACCCGCTGAACTTAAGCATATCAATAAGCCGGAGGAA

b ITS4, Serial number:
AGASTGGATCCTACTGATCCGAGGTCACCTGGAAAAAATGGTTGGAAAACGTCGG-

CAGGCGCCGGCCAATCCTACAGAGCATGTGACAAAGCCCCATACGCTCGAGGATCGGAC-

GCGGTGCCGCCGCTGCCTTTCGGGCCCGTCCCCCCGGAGAGGGGGAC-

GGCGACCCAACACACAAGCCGGGCTTGAGGGCAGCAATGACGCTCGGACAGGCATGCCCCCCG-

GAATACCAGGGGGCGCAATGTGCGTTCAAAGACTCGATGATTCACTGAATTCTG-

CAATTCACATTAGTTATCGCATTTCGCTGCGTTCTTCATCGATGCCGGAACCAAGA-

GATCCATTGTTGAAAGTTTTAACTGATTGCATTCAATCAACTCAGACTGCACGCTTTCAGA-

CAGTGTTCGTGTTGGGGTCTCCGGCGGGCACGGGCCCGGGGGG-

CAAAGGCGCCCCCCCGGCGGCCGACAAGCGGCGGGCCCGCCGAAGCAACAGGGTATAATAGA-

CACGGATGGGAGGTTGGGCCCAAAGGACCCGCACTCGGTAATGATCCTTCCGCAGGTTCAC-

CTACGGAAACCTTGTTACGACTTTTACTTCCTCAAGGGRAMCCAAGRA

Description	Scientific Name	Max Score	Total Score	Query Cover	E value	Per. Ident	Acc. Len	Accession
Aspergillus tubingensis isolate 132 small subunit ribosomal RNA gene, partial sequence; internal transcribed spacer 1, 5.8S ribosomal RNA gene, and internal transcribed spacer 2, complete sequence; and large subunit ribosomal RNA gene, partial sequence	Aspergillus tubingensis	1101	1101	98%	0.0	99.83%	609	KU847852.1
Aspergillus niger isolate 127 internal transcribed spacer 1, partial sequence; 5.8S ribosomal RNA gene and internal transcribed spacer 2, complete sequence; and large subunit ribosomal RNA gene, partial sequence	Aspergillus niger	1099	1099	98%	0.0	99.83%	608	KU847850.1

c ITS1, Serial number:
AGSCAGCGCTTCCGTAGGTGACCTGCGGAGGATCATTACAGAGTTCATGCCCGAAAGGG-

TAGACCTCCCACCCTTGTGTATTATTACTTTGTTGCTTTGGCGAGCTGCCTTCGGGCCTT-

GTATGCTCGCCAGAGAAAACCAAAACTCTTTTTATTAATGTCGTCTGAGTACTATATAA-

TAGTTAAAACTTTCAACAACGGATCTCTTGGTTCTGGCATCGATGAAGAAC-

GCAGCGAAATGCGATAAGTAATGTGAATTGCAGAATTCAGTGAATCATCGAATCTTTGAAC-

GCACATTGCGCCCCTTGGTATTCCGGGGGGCATGCCTGTTCGAGCGTCATTTCAACCCTCAA-

GCTTAGCTTGGTATTGAGTCTATGTCAGTAATGGCAGGCTCTAAAATCAG-

TGGCGGCGCCGCTGGGTCCTGAACGTAGTAA-

TATCTCTCGTTACAGGTTCTCGGTGTGCTTCTGCCAAAACCCAAATTTTTCTATGGTTGAC-

CTCGGATCAGGTAGGGATACCCGCTGAACTTAAGCATATCAAARCSCGGAGGAAA

c ITS4, Serial number:
TRTGGAGCTACCTGATCCGAAGGTCACCATTAGAAAAATTTGGGTTTTGGCAGAAGCACAC-

CGAGAACCTGTAACGAGAGATATTACTACGTTCAGGACCCAGCGGCGCCGCCACTGATTTTA-

GAGCCTGCCATTACTGACATAGACTCAATACCAAGCTAAGCTTGAGGGTTGAAATGAC-

GCTCGAACAGGCATGCCCCCCGGAATACCAAGGGGCGCAATGTGCGTTCAAAGAT-

TCGATGATTCACTGAATTCTGCAATTCACATTACTTATCG-
CATTTCGCTGCGTTCTTCATCGATGCCAGAACCAAGAGATCCGTTGTTGAAAGTTTTAACTAT-
TATATAGTACTCAGACGACATTAATAAAAAGAGTTTTGGTTTTCTCTGGCGAGCATA-
CAAGGCCCGAAGGCAGCTCGCCAAAGCAACAAAGTAATAATACACAAGGGTGGGAGGTC-
TACCCTTTCGGGCATGAACTCTGTAATGATCCTTCCGCAGGTTCACCTACGGAAACCTT-
GTTACGACTTTTACTTCCTCAAGRGGACARRRACC

Description	Scientific Name	Max Score	Total Score	Query Cover	E value	Per. Ident	Acc. Len	Accession
Botrytis cinerea isolate R10 small subunit ribosomal RNA gene, partial sequence; internal transcribed spacer 1, 5.8S ribosomal RNA gene, and internal transcribed spacer 2, complete sequence; and large subunit ribosomal RNA gene, partial sequence	Botrytis cinerea	955	955	95%	0.0	99.43%	536	MK513827.1

Figure S2. Identification of dominant pathogenic fungi isolated from grape. (a: *P. expansum*, b: *A. niger* and c: *B. cinerea*.)

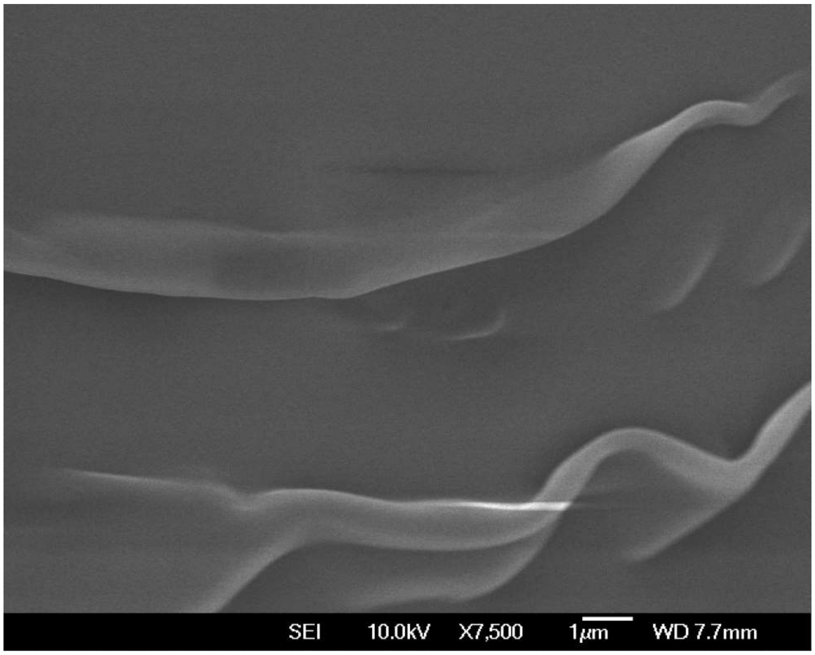

Figure S3. SEM image of the CS gel.

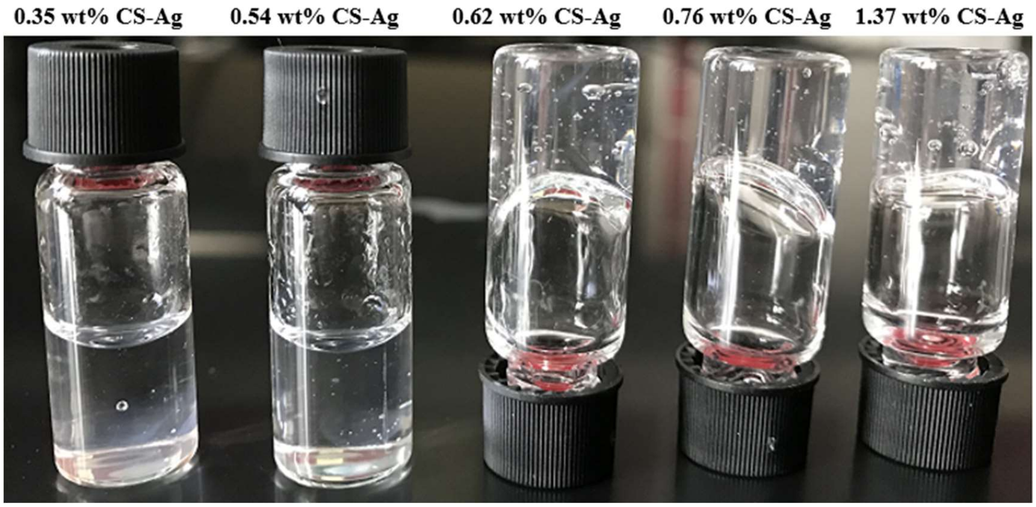

Figure S4. The CGC of CS-Ag complex hydrogel.

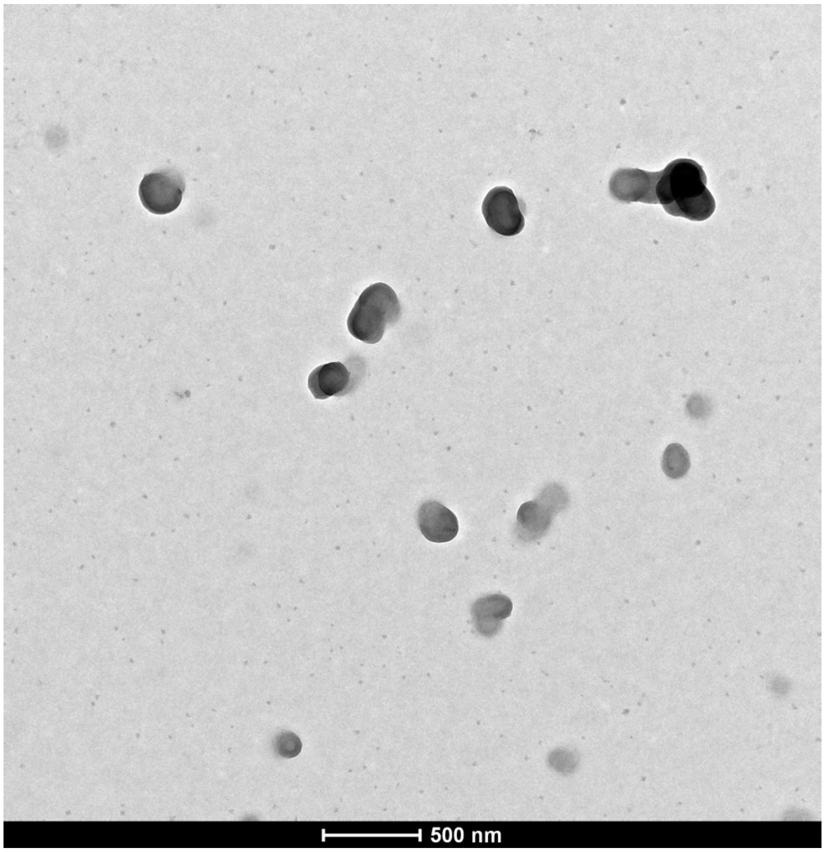

Figure S5. TEM image of AgNPs.

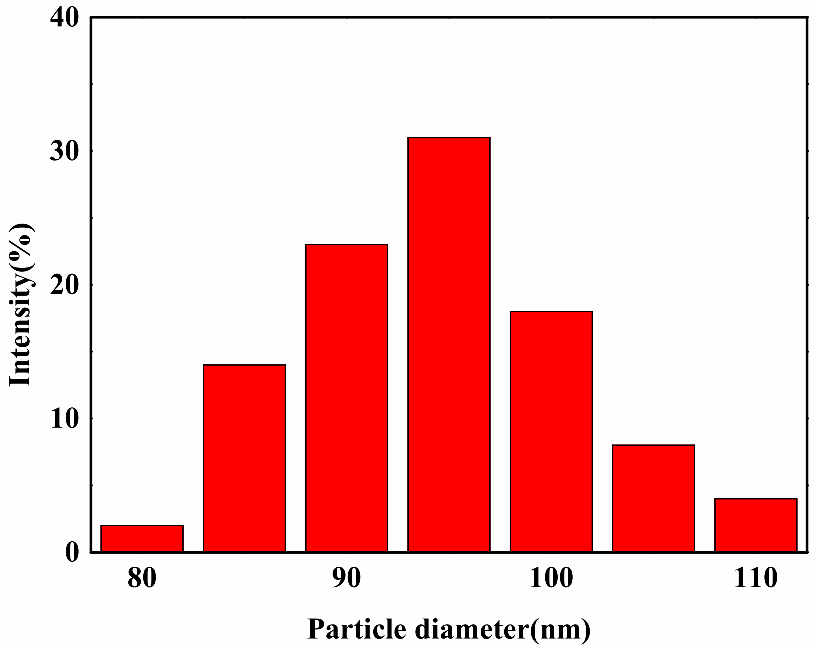

Figure S6. The size distribution of AgNPs.

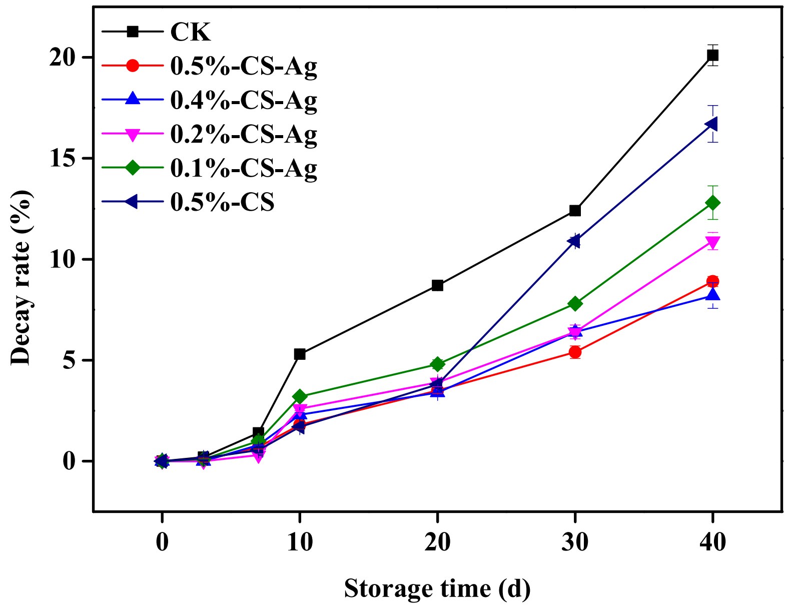

Figure S7. CS-Ag hydrogel effect on decay rate of grape during postharvest.

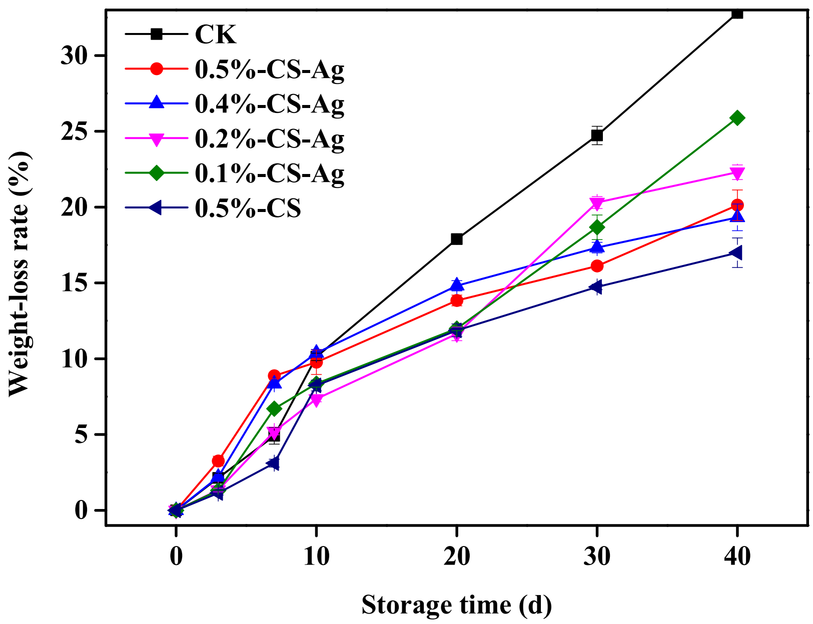

Figure S8. CS-Ag hydrogel effect on weight loss rate of grape during postharvest.

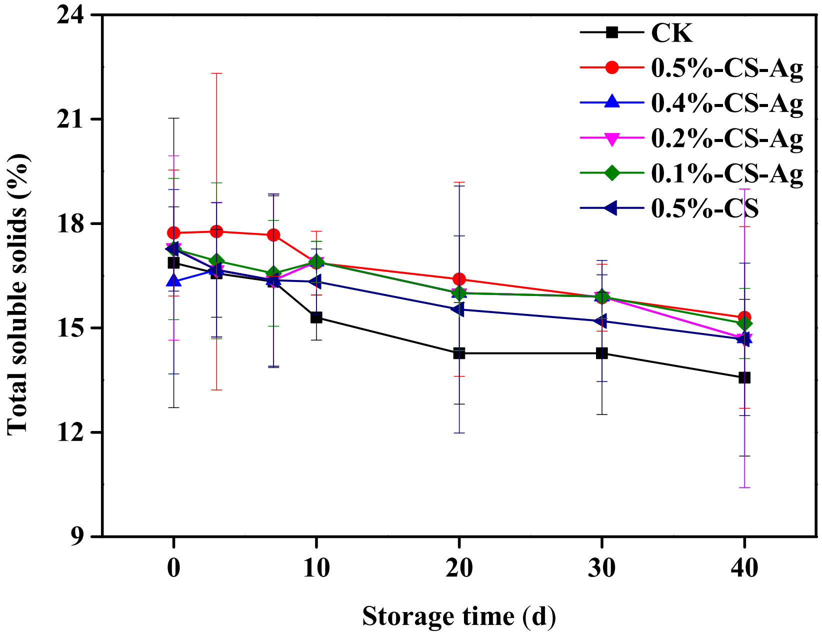

Figure S9. CS-Ag hydrogel effect on total soluble solids of grape during postharvest.

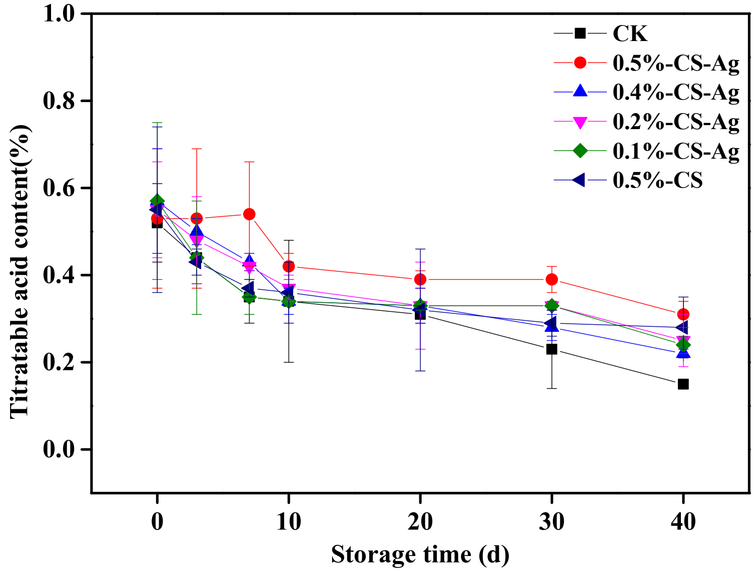

Figure S10. CS-Ag hydrogel effect on titratable acids of grape during postharvest.

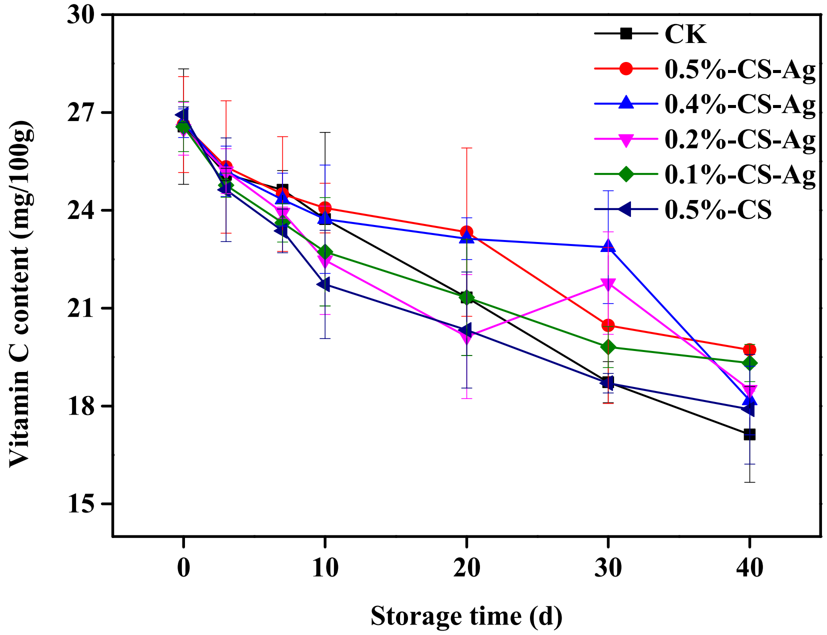

Figure S11. CS-Ag hydrogel effect on Vitamin C of grape during postharvest.
